# *Atg16l1* and *Xbp1* cooperatively protect from transcription-associated mutagenesis and small intestinal carcinogenesis

**DOI:** 10.1038/s41388-025-03591-x

**Published:** 2025-10-07

**Authors:** Nassim Kakavand, Hang Xiang, Georg Laue, Taous Mekdoud, Lina Welz, Miguel Gomes Silva, Joana P. Bernardes, Go Ito, Silke van den Bossche, Julia Kugler, Florian Tran, Alexander Ossysek, Simon Imm, Finn Hinrichsen, Moritz Jesinghaus, Arthur Kaser, Richard Blumberg, Timon E. Adolph, Stefan Schreiber, Markus Tschurtschenthaler, Philip Rosenstiel, Konrad Aden

**Affiliations:** 1 Institute of Clinical Molecular Biology, Christian-Albrechts-University and University Hospital Schleswig-Holstein, Campus Kiel, Kiel, Germany; 2 Department of Internal Medicine I., Christian-Albrechts-University and University Hospital Schleswig-Holstein, Campus Kiel, Kiel, Germany; 3 Center for Translational Cancer Research (TranslaTUM), Klinikum rechts der Isar, School of Medicine and Health, Technical University of Munich, Munich, Germany; 4 Institute of Pathology, University Hospital Marburg, Marburg, Germany; 5 Division of Gastroenterology and Hepatology, Department of Medicine, Addenbrooke’s Hospital, University of Cambridge, Cambridge, UK; 6 Gastroenterology Division, Department of Medicine, Brigham and Women’s Hospital, Harvard Medical School, Boston, MA USA; 7 Department of Internal Medicine I, Gastroenterology, Hepatology, Endocrinology & Metabolism, Medical University of Innsbruck, Innsbruck, Austria; 8 Division of Translational Cancer Research, German Cancer Research Center (DKFZ) and German Cancer Consortium (DKTK), Heidelberg, Germany; 9 Chair of Translational Cancer Research and Institute of Experimental Cancer Therapy, Klinikum rechts der Isar, School of Medicine and Health, Technical University of Munich, Munich, Germany

**Keywords:** Colorectal cancer, Mechanisms of disease

## Abstract

*Atg16l1* plays a critical role in autophagy, and *Xbp1* is part of the endoplasmic reticulum (ER) homeostasis. Both, *Atg16l1* and *Xbp1* are known risk genes for inflammatory bowel disease (IBD). Previous studies have shown that dysfunctional *Atg16l1* and *Xbp1* are epithelial-derived drivers of small intestinal inflammation. Despite a clear link between Crohn’s disease and small intestinal adenocarcinoma, the molecular impact of autophagy and ER stress in this malignant transformation is not known. Using a model of impaired ribonucleotide excision repair (RER), a key homeostatic repair mechanism in highly proliferative cells, we investigated the impact of *Atg16l1* on epithelial DNA damage responses and small intestinal carcinogenesis with and without functional ER homeostasis. We used conditional mouse models for deficient RER (*Rnaseh2b*^ΔIEC^), bearing a co-deletion of disrupted autophagy (*Atg16l1/Rnaseh2b*^ΔIEC^) or ER stress resolution (*Xbp1/Rnaseh2b*^ΔIEC^), and triple-conditional knock-out mice for both, *Xbp1* and *Atg16l1* (*Atg16l1/Xbp1/Rnaseh2b*^ΔIEC^). We assessed the degree of DNA damage and the incidence of small intestinal carcinoma. We report that defective epithelial RER induces autophagy, and that dysfunctional autophagy increases RER-induced DNA damage and causes the loss of RER-induced proliferative arrest but no spontaneous carcinogenesis in the gut. We demonstrate that dysfunctional *Atg16l1* drastically increases the incidence of spontaneous intestinal adenocarcinomas in mice with defective epithelial RER and impaired ER homeostasis. We provide experimental evidence that the same epithelial mechanisms suppressing gut inflammation also critically protect from small intestinal carcinogenesis. Our findings set a molecular framework for the increased risk of intestinal carcinogenesis in patients with IBD, which links perturbations of ER homeostasis and autophagy defects with accumulating DNA damage.

**Figure:**
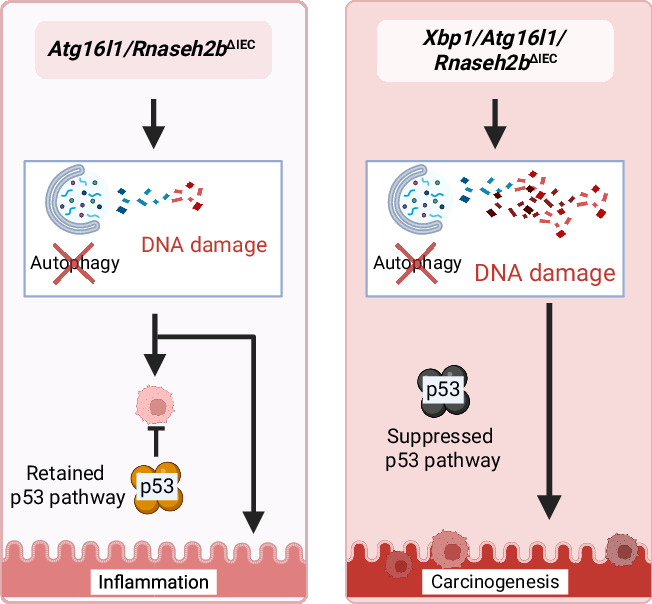
In a model of transcription-associated mutagenesis, deficiency of the IBD risk gene *Atg16l1* does not induce small intestinal cancer. In contrast, double deficiency of *Xbp1* and *Atg16l1* drives spontaneous tumor formation highlighting a cooperative role of *Xbp1* and *Atg16l1* in tumor suppression.

In a model of transcription-associated mutagenesis, deficiency of the IBD risk gene *Atg16l1* does not induce small intestinal cancer. In contrast, double deficiency of *Xbp1* and *Atg16l1* drives spontaneous tumor formation highlighting a cooperative role of *Xbp1* and *Atg16l1* in tumor suppression.

## Introduction

Inflammatory bowel diseases (IBD) are chronic inflammatory diseases of the gastrointestinal tract. The two disease subtypes, Crohn’s disease (CD) and ulcerative colitis (UC), differ in their anatomical locations of disease manifestation, the nature of mucosal lesions, and the disease behavior. IBD is associated with a 1.7-fold increase in intestinal cancer compared to the healthy population, which is mainly driven by disease extent and disease duration [1]. Although inflammation-associated carcinogenesis is established as a long-term consequence of IBD, the molecular origin remains poorly understood. Chronic inflammation can shape the mutational landscape of IECs, and it has been shown that somatic mutations, such as insertion-deletion mutations (indels) are more prevalent even in the non-neoplastic mucosa of IBD-affected individuals [2]. Transcription-associated mutagenesis has recently been attributed to the development of somatic cancers in eukaryotes [3]. It describes the mechanism of compensatory TOP1-mediated DNA repair at sites of increased frequency of genome-embedded ribonucleotides, resulting in increased indel formation. Mechanistically, genome-embedded ribonucleotides are the consequence of impaired ribonucleotide excision repair, which removes faulty DNA-incorporated ribonucleotides during every round of cellular replication via the enzyme RNase H2 [4, 5]. We have recently shown that conditional deletion of *Rnaseh2b* in murine intestinal epithelial cells (*Rnaseh2b*^ΔIEC^) leads to chronic DNA damage and p53-dependent suppression of stem cell proliferation. In addition, linking chronic inflammation to the acquisition of somatic mutations, we have shown that *Rnaseh2b*^ΔIEC^ mice present with histological intestinal enteritis, which further substantiates the link between chronic DNA damage and IBD pathophysiology [6]. Several lines of evidence point to a crucial regulatory role of ER stress and autophagy in the context of IBD, as defects for both, ER stress and autophagy, have been shown to instigate spontaneous small intestinal inflammation in mouse models of IBD [7–9]. Along this line, it has been further shown that ER stress and autophagy cooperate through a cross-compensatory mechanism to resolve stress conditions, such as the increased accumulation of unfolded proteins, via induction of autophagy [10]. Despite the clear genetic link between these two homeostatic factors on the manifestation of small intestinal inflammation, it is not known whether ER stress and autophagy are cooperatively needed to prevent inflammation-associated carcinogenesis. This is of particular importance given the rising incidence of small bowel adenocarcinoma in patients with Crohn’s disease [11, 12]. In this context, stricturing and penetrating ileal Crohn’s disease have been shown to be associated most strongly with the manifestation of small bowel adenocarcinoma (SBA), underscoring the relevance of unresolved mucosal inflammation as an independent trigger of carcinogenesis. Importantly, it has been previously shown that the genomic landscape of SBA is significantly different in IBD-associated SBA, compared to non-IBD associated, with mutations within the p53 pathway being affected in nearly 70% of all IBD-associated SBA [13]. Initial indications for a molecular interaction between chronic DNA damage and IBD were recently shown in the context of the IBD risk gene *X-box binding protein 1* (*XBP1*) [14]. In the context of impaired RER, mice with a conditional double deficiency of *Xbp1* and *Rnaseh2b* (*Xbp1/Rnaseh2b*^ΔIEC^) displayed increased epithelial proliferation, increased intestinal inflammation and 50% of *Xbp1/Rnaseh2b*^ΔIEC^ mice developed spontaneous manifestation of intestinal adenocarcinoma [15]. Here, we set out to investigate the role of *ATG16L1* and *XBP1* in coordinating cellular responses towards dysfunctional RER and subsequent DNA damage and carcinogenesis. Furthermore, we examined whether the functioning interplay between *ATG16L1* and *XBP1*, which is known to contribute to sporadic Crohn-like enteritis in mouse models of IBD, might be involved in transcription-associated mutagenesis and the manifestation of intestinal tumors. Using a genetic model of deficient RER, we show that *Atg16l1* deficiency increases the incidence of spontaneous intestinal carcinogenesis to 90% of mice that are simultaneously challenged by *Xbp1*-deficiency [8]. By contrast, epithelial *Atg16l1* deficiency alone impairs the DNA damage response but does not lead to spontaneous carcinogenesis. Our study sheds light on the critical interplay between autophagy and ER stress on transcription-associated mutagenesis and implies that these mechanisms might be relevant in the manifestation of small bowel cancer in IBD patients.

## Materials and Methods

All authors had access to the study data and had reviewed and approved the final manuscript.

### Mice

Intestinal epithelial cell-specific knockout mice were generated by means of the *Villin*-Cre/loxP system. *Atg16l1*^fl/fl^, *Xbp1*^fl/fl^, and *Rnaseh2b*^fl/fl^ mice were generated as described previously [8, 14, 16]. These mice were crossed with *Villin-Cre*^+^ mice to generate *Atg16l1*^ΔIEC^ (*Villin-Cre*^+^; *Atg16l1*^fl/fl^), *Xbp1*^ΔIEC^ (*Villin-Cre*^+^; *Xbp1*^fl/fl^), *Rnaseh2b*^ΔIEC^ (*Villin-Cre*^+^; *Rnaseh2b*^fl/fl^) mice, as well as *Atg16l1/Rnaseh2b*^ΔIEC^ (*Villin-Cre*^+^; *Atg16l1*^fl/fl^, *Rnaseh2b*^fl/fl^), *Xbp1/Rnaseh2b*^ΔIEC^ (*Villin-Cre*^+^; *Xbp1*^fl/fl^, *Rnaseh2b*^fl/fl^) and *Atg16l1/Xbp1/Rnaseh2b*^ΔIEC^ (*Villin-Cre*^+^; *Atg16l1*^fl/fl^, *Xbp1*^fl/fl^, *Rnaseh2b*^fl/fl^) mice. Gene expression analysis of the tail was performed to determine the variant of the indicated genes. For experiments with tissue derived from these mice, we confirmed the correct genotype by carrying out gene expression analysis or western blot in each experiment. We used young (8 weeks–20 weeks) or aged (40–65 weeks) mice that were maintained in a specific pathogen–free facility, and the quarterly health report did not indicate the presence of pathogenic species. Mice were separated by genotypes and were provided with food and water *ad libitum* and maintained in a 12 h light–dark cycle under standard conditions at Kiel University. For experiments, age-matched female and male animals were used. Procedures involving animal care were conducted conform to national and international laws and policies with appropriate permission. All experiments were performed in accordance with the guidelines for animal care of Kiel University (Animal Vote V 241 - 61544/2017 (69-5/17) and V 242 - 29834/2016 (52-5/16)).

### Bromodeoxyuridine (BrdU) Administration in Mice

For the immunohistological analysis of the intestinal proliferation, we used *inter alia* the BrdU assay. Mice were peritoneally injected with a solution of 10 µg BrdU per µl PBS. This solution was injected weight-adapted with a ratio of 10 µl per 1 g body weight, to achieve a concentration of 100 µg BrdU per g body weight. After incubation for 90 minutes, mice were sacrificed, and histological slides were generated (see below).

### Histopathological preparation of murine intestinal tissue

Postmortem, the small intestine and colon were excised, cut open longitudinally, and rinsed carefully in a Petri dish filled with PBS. Swiss rolls from the ileum and the colon were generated by rolling up each section from the distal to the proximal part and were fixed in formalin solution (10% neutral buffered). Paraffin sections were cut in 5 µm slides and then stained with H&E or other specific staining. For the detection of DNA damage, we used Anti-Phospho-Histone H2A.X antibodies. For the detection of apoptotic and necrotic cells, we used TUNEL (TUNEL-POD, Roche). For the detection of proliferative cells, we used Anti-BrdU and Anti-Ki-67 antibodies. Find detailed information about antibodies in Supplementary Table 1 and 2.

### Histopathological analysis of DNA damage, cell death and proliferation

For the histopathological analysis of the intestinal tissue, we used paraffin-embedded rolls of the intestine and prepared them as aforementioned. The sample size for each genotype was based on prior studies and preliminary experiments and were deemed sufficient to detect biologically meaningful differences between genotypes [6, 15]. For counting, we used a Zeiss Axio Vert.A1 microscope. For both the small intestine as well as the colon, we counted a minimum of 24 evenly distributed crypts along the whole intestinal roll with a focus on the bottom 12 cells of each crypt. Stained slides were randomly shuffled and coded to ensure blinding. The analysis was carried out by two independent blinded observers. Only results were considered significant that showed homogenous changes between groups in two independent observations. After statistical analysis, representative pictures were made for each genotype with the aid of AxioVision LE (Zeiss) software.

### Histopathological Scoring of Enteritis

Histological scoring of enteritis was performed as described previously [8]. The score displays the combined score of inflammatory cell infiltration and tissue damage and was performed in a blinded fashion by two independent observers. Inflammation levels were quantified by assessment of the following parameters: mononuclear cell infiltrate, crypt hyperplasia, epithelial injury/erosion, polymorphonuclear cell infiltrate, and transmural inflammation. Each parameter was graded as follows: non-existent = 0 points, slight = 1 point, moderate = 2 points, severe = 3 points.

### Histopathological analysis of murine intestinal tumors

Macroscopically identifiable tumors of the small intestine were chosen for histopathological analysis. To guarantee comparability, we selected the largest (diameter) tumor of each mouse. The tumors were stained with H&E and classified in analogy to the recent WHO classification of tumors of the Digestive System (fifth edition) [17]. Invasive adenocarcinomas were graded as “low-grade” and “high-grade” according to the extent of gland formation. The grade of Dysplasia/Intraepithelial Neoplasia (IEN) of small intestinal adenomas was classified into low- and high-grade Dysplasia/Intraepithelial Neoplasia IEN based on the degree of architectural complexity, extent of nuclear stratification, and severity of abnormal nuclear morphology.

### cDNA synthesis and gene expression analysis

Using the RNeasy Kit (Qiagen), mRNA was isolated from ModeK cells and organoids washed in PBS or from snap-frozen tissue. cDNA was synthesized using the Maxima H Minus First Strand cDNA Synthesis Kit (Thermo Fisher Scientific) according to the manufacturer’s protocol. To examine gene expression, cDNA samples were subjected to quantitative reverse transcription polymerase chain reaction using either SYBR Green or TaqMan assays purchased from Applied Biosystems, Reactions were carried out on the Applied Biosystems 7900HT Fast Real-Time PCR System (Applied Biosystems), and relative transcript levels were determined utilizing either *Actb* or *Gapdh* as housekeepers. Deploying PrimerBLAST-Software (NIH), primer sequences were designed, while TaqMan probes were derived from Thermo Fisher Scientific. Find detailed information about TaqMan probe IDs and SYBR Green primers in Supplementary Tables 3 and 4.

### Murine intestinal epithelial ModeK cells

XBP1-deficient ModeK cells were generated by transfection with a lentivirus-expressed shRNA against *Xbp1* as described previously [18]. Knockdown was validated by qPCR analysis. Cells were cultivated at 37 °C with DMEM GlutaMAX^TM^ medium (Gibco) with 10% FCS (Merck Millipore), 1% HEPE (1 M) (Gibico), 1% NEAA (100X) (Gibico). The medium was exchanged every other day. For in vitro transfection, Viromer© Blue (Lipocalyx) was used according to the manufacturer’s protocol. siRNA against *Atg16l1* (#GS77040) and *Rnaseh2b* (#GS67153) was derived from Qiagen and used at a concentration of 10 μM.

### Immunofluorescence staining of adherent cells

Cells were washed with PBS and fixed with 2% (w/v) formalin for 30 min at room temperature. After washing two times with PBS, permeabilization was performed by incubation for 3 min with 1% (w/v) BSA and 0.1% (v/v) Triton X-100 in PBS. We blocked for 1 h with 5% (w/v) BSA. The primary antibody (Supplementary Table 1) was incubated with a dilution of 1:200 overnight at 4 °C with 5% (v/v) goat serum diluted in PBS. After washing three times, incubation with the secondary antibody (Supplementary Table 2) was performed for 1 h at room temperature (dilution 1:500 in PBS with 5% (w/v) BSA). Counterstaining was performed with DAPI (1:40,000 in PBS) for 10 min. Afterwards, the chamber was removed from the plate. Slides were washed in *aqua bidest* and then dried. DAKO mounting medium (Agilent Technologies) was put on the slides together with a cover slip.

### Generation of CRISPR/Cas9-guided deletion of *Atg16l1* in ModeK Cells

A CRISPR/Cas9 vector (Thermo Fisher Scientific) was created using GeneArt™ CRISPR Nuclease Vector with CD4 Enrichment Kit according to the manufacturer’s instructions, using a dsDNA oligo targeting exons 1 and 3 of *Atg16l1* (forward: 5’-GTCACATCGCGGAGGAACTGGTTTT-3’, reverse: 3’-CAGTTCCTCCGCGATGTGACCGGTG-5’). The plasmid was validated by Sanger sequencing. The vector was transfected with Lipofectamine 3000 into ModeK cells, and clonal cell lines were generated. Knockout clones were selected by verifying the absence of ATG16L1 protein by Western blot analysis.

### Immunoblot analysis

Cells were lysed using sodium dodecyl sulfate (SDS)–based DLB buffer with 1% Halt Protease inhibitor cocktail (Thermo Fisher Scientific), heated at 95 °C for 5 min, and exposed to ultra-sonication for 5 seconds twice. Lysates were centrifuged at 16,000 g for 15 min at 4 °C to remove cell remnants. For protein extraction of organoids, Matrigel was removed by several centrifugation steps at 4 °C, followed by lysis as described earlier. Afterward, equal amounts of lysates containing Laemmli buffer were electrophoresed on 12% polyacrylamide gels under standard SDS-PAGE conditions before being transferred onto polyvinylidene fluoride membranes (GE Healthcare). Protein-loaded membranes were blocked with 5% milk in Tris-buffered saline and Tween 20 (TBST) before being incubated with primary antibody at 4 °C overnight, followed by incubation with a horseradish peroxidase-conjugated secondary antibody for 1 h at indicated concentrations. Proteins were detected using the Amersham ECL Prime Western Blot Detection Reagent (GE Healthcare).

### Cultivation of murine intestinal organoids

Isolation of crypts and initiation of cultivation of intestinal organoids was performed as described previously [19]. Matrigel (BD Bioscience) embedded crypts were either cultivated in Intesticult Organoid Growth Medium (Intesticult; STEMCELL Technologies) or ENR conditioned organoid medium (epidermal growth factor / Noggin / R-spondin1 [20]. For passaging, mature organoids (7–10 days after the last passage) were transferred to a 15 ml tube and mechanically divided using a 1000 µl pipet. For experiments, organoids were used 3 - 6 days after passage.

### Quantification of organoid-forming capacity

On day three after passage, the organoids were resuspended in TrypLE Express (Thermo Fisher Scientific), transferred to a 15 ml tube, and incubated in a 37 °C water bath for 10 min. Before and after the water-bath the content was resuspended with a 1000 µl pipette tip. The resulting single cell suspension was centrifuged at 400 *g* for 3 min. The resulting pellet was resuspended in 500 µl DMEM/F-12 (Thermo Fisher Scientific). From this, 20 µl were taken and the number of single cells was determined via the Cellometer Auto T4 Plus (Heraeus). Per well 10,000 cells were seeded out in a 40 µl Matrigel (BD Bioscience) droplet on a 24-well plate. Medium was added after polymerizing the Matrigel for >10 min in the incubator at 37 °C. The amount and Diameter of organoids were assessed after 3 days and 6 days. The diameter was determined by taking pictures of the biggest organoids per well with the Axio Observer A1 (ZEISS) microscope and measuring with the AxioVision SE64 Rel. 4.9.1 (ZEISS) software.

### RNA sequencing

Total RNA was extracted from WT (*n* = 4) and *Atg16l1*^ΔIEC^ (*n* = 4), *Rnaseh2b*^ΔIEC^ (*n* = 4), and *Atg16l1*/*Rnaseh2b*^ΔIEC^ (*n* = 4) small intestinal organoids using RNeasy Mini Kit (QIAGEN), following the manufacturer’s protocol. Two samples from the *Rnaseh2b*^ΔIEC^ group were excluded from analysis due to a quality issue (read counts), resulting in a final *n* = 2 for this group. Sequencing was performed on HiSeq3000 (Illumina, San Diego, CA, USA) using the TruSeq stranded mRNA protocol (Illumina). A 1 ×50 bp single-read run was performed. Preprocessing was performed with cutadapt for removal of low-quality and adapter sequences [21]. Reads were aligned to the *Mus musculus* genome (GRCm38) with TopHat2 [22]. Gene expression values were computed with HTSeq [23]. The Bioconductor package DESeq2 was used to analyze differential gene expression and principal component analysis (PCA) [24]. The Venn diagram package was used for the generation of Venn diagrams [25]. Gene Ontology terms were obtained within the category of biological processes from the InnateDB (www.innatedb.com) [26]. Transcription factor binding site analysis was performed using the Bioconductor package TFBSTools [27].

### Quantification of organoid viability

Organoids were dissociated by pipetting approximately 100 times and evenly seeded into a 24-well plate. After 72 h of culture by ENR medium containing DMSO or 1 μM rapamycin, CellTiter-Glo® 3D reagent (Promega) was added according to the manufacturer’s instructions. Luminescence was then measured using a microplate reader (Tecan).

### Organoid roundness analysis

Organoids images from Day4-Day5 after seeding were taken by microscopy. Bright-field images were analyzed by segmenting individual organoids using an AI tool (Segment Anything Model), and once the mask coordinates were generated, organoid roundness was calculated using the following function: Roundness=4π⋅Area/(Perimeter)^2^. Only isolated organoids above a defined size threshold were included. Group differences were assessed using the unpaired Student’s *t*-test [28].

### Comet assay

ModeK cells were transfected with control siRNA or siRNA against *Atg16l1* after 24 h of cultivation. The cells were stimulated with 2.5 µM AraC after another 24 h. Afterward, cells were harvested and quantified by a cell counter (LUNA II LUNA-II™, Logos Biosystem). The following assessment of DNA damage was conducted using a comet assay® (4250-050-ESK, R&D Systems) according to the manufacturer’s recommended protocol. The LMA (low-melting-point agarose) gel was melted at 90 °C using a water bath. Once melted, it was incubated at 37 °C. Concurrently, cells were mixed with the LMA gel, and 100 μl of the mixture was dropped onto a CometSlide, followed by flattening using pipette tips. After allowing the gel to cool at 4 °C for 10 min, it was immersed in lysis buffer and kept at 4 °C overnight. The cells embedded in the gel were subjected to an alkaline unwinding solution for 20 min. Electrophoresis was performed at 21 volts for 30 min using an alkaline running buffer maintained at 4 °C. The slides were washed twice with deionized water and once with 70% ethanol solution after electrophoresis. The slides were stained with SYBR Gold (Invitrogen™ SYBR™ Gold Nucleic Acid Gel Stain, S1194) for 10 min in the dark. After staining, the slides were rinsed with deionized water and dried at 37 °C.

### Ethics approval and consent to participate

All in-vivo mouse models were performed in accordance with the relevant guidelines with the guidelines for animal care of Kiel University and upon approval of the state authorities (Ministerium für Energiewende, Klimaschutz, Umwelt und Natur, Schleswig-Holstein, Germany). No human biomaterial was used for this study.GoAAr

### Statistical analysis

The statistical analysis was performed using GraphPad Prism, version 8.0.2 (GraphPad Software, Inc.) All statistical data were presented as means ± SEM. Overall survival (OS) was analyzed using Kaplan–Meier survival analysis. Group differences in survival were assessed using both the log-rank test and the Gehan–Breslow–Wilcoxon test. Normality was assessed using the Shapiro-Wilk test. Statistical comparisons between two independent groups were performed using an unpaired Student’s *t*-test. Homogeneity of variance was assessed using an F-test automatically performed by GraphPad Prism, and Welch’s correction was applied when variances were unequal. Differences between genotypes were analyzed using the Mann–Whitney U test. All statistical tests were two-sided, and p-values < 0.05 were considered statistically significant. Further statistical information can be found in the figure legends.

## Results

### *Atg16l1* is required for DNA damage-induced autophagy in intestinal epithelial cells

**Fig. 1:**
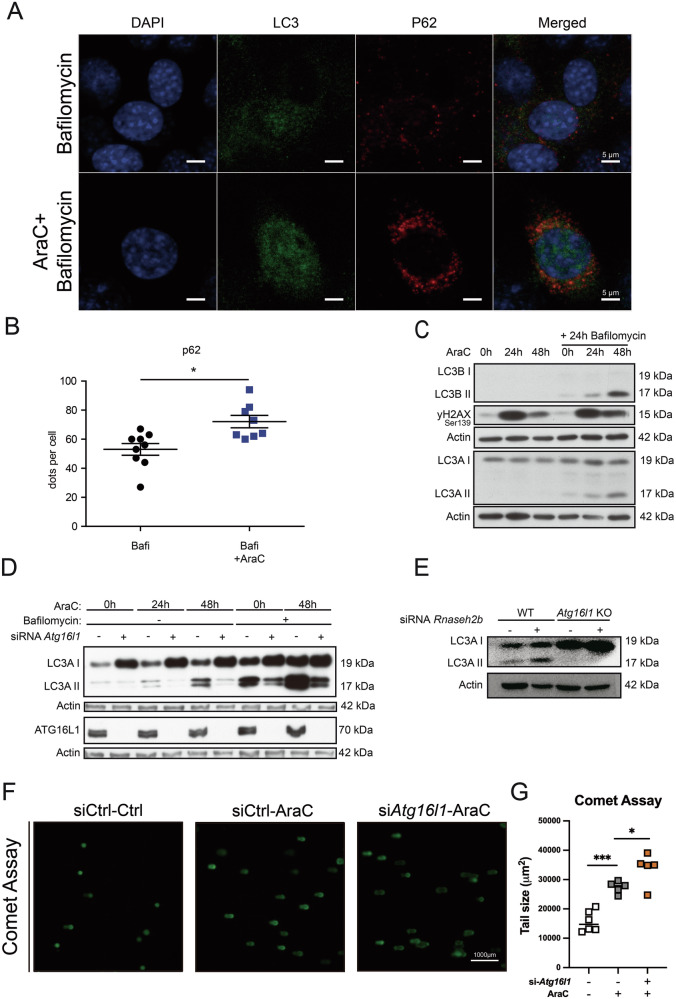
*Atg16l1* mediates DNA damage-induced autophagy in intestinal epithelial cells. **A**, **B** Immunofluorescence staining of Mode-K cells showed induced accumulation of p62 upon AraC treatment. Mode-K cells were stained with LC3 and p62/SQSTM1 antibodies after stimulation with 2.5 μM cytarabine for 24 h or left unstimulated. Additionally, co-stimulation with 100 nM bafilomycin for 2 h before fixation and staining was performed. Representative images (**A**) of immunofluorescence-stained cells were taken. Formed p62/SQSTM1 dots per cell (**B**) were counted in randomly chosen Mode-K cells (*n* > 8). **C** AraC treatment increased LC3I/II conversion. Representative image of 4 independent experiments. Statistical comparison of the intensity of LC3 II bands from cells stimulated with bafilomycin and with versus without 48 h AraC was performed using the Wilcoxon matched-pairs signed rank test (*p* = 0,125). Proteins were extracted from Mode-K cells stimulated with 2.5 μM AraC for 24 and 48 h or left unstimulated. Additionally, the same setup was stimulated with 5 nM bafilomycin for 24 h. **D** Knockdown of *Atg16l1* severely impaired DNA damage-induced autophagy. Representative image of 3 independent experiments. Statistical comparison of the intensity of LC3 II bands from cells stimulated with bafilomycin, 48 h AraC treatment, and with versus without siRNA-guided knockdown of *Atg16l1* was performed using the Wilcoxon matched-pairs signed rank test (*p* = 0,25). Proteins were extracted from Mode-K cells. The cells were transfected either with siRNA against *Atg16l1* or, as a control, with scrambled siRNA. They were treated with 2.5 μM AraC for 24 h, 48 h, or left unstimulated. One group of unstimulated cells and 48 h AraC-stimulated cells was additionally co-stimulated with 10 nM bafilomycin. **E** Increased LC3I/II conversion, after siRNA-mediated downregulation of *Rnaseh2b*. Representative image of 2 independent experiments. Control Mode-K cells and CRISPR/Cas mediated *Atg16l1* KO Mode-K cells were treated with mock siRNA or siRNA *Rnaseh2b* for 24 h. **F**, **G** A Comet assay shows absence of Atg16l1 leads to an increased amount of DNA damage. Following 24 h of siRNA transfection, cells were stimulated with 2.5 µM AraC for 24 h, then a comet assay was conducted. The comet- and tail size was quantified. Tail size = comet size (total size) – head size (Average nuclear without tail), negative values were counted as 0. Data are shown as Median and were tested for statistical significance using an unpaired *t*-test. * *p* < 0.05, *** *p* < 0.001. Scale bar= 1000 μm. Ctrl = control, AraC = cytarabine, Bafi = bafilomycin.

We have previously shown that intestinal epithelial deficiency of RNase H2, the rate-limiting enzyme of the ribonucleotide excision repair machinery, instigates chronic DNA damage [6]. First, we delineated a role for ATG16L1-mediated autophagy during DNA damage. To do so, we assessed whether DNA damage evoked by cytarabine (AraC) induces autophagy in intestinal epithelial cells (IECs, ModeK murine small intestinal cell line) with or without exposure to bafilomycin, which inhibits the V-ATPase-dependent acidification and autophagosome-lysosome fusion. We conducted immunofluorescence staining of ModeK cells for p62, a surrogate of autophagic flux, as p62 might accumulate upon DNA damage in the presence of bafilomycin. Indeed, we observed that AraC-induced DNA damage led to the accumulation of p62 (Fig. 1A, B). This was paralleled by increased LC3-I/II conversion (Fig. 1C), indicating that DNA damage induced autophagy. To corroborate this observation, we performed siRNA-guided silencing of *Atg16l1*, a key homeostatic regulator of the autophagy machinery, which is implicated in the pathogenesis of IBD. Indeed, *Atg16l1* silencing decreased LC3AI/II conversion upon AraC treatment, indicating that ATG16L1 is causally involved in mediating the autophagic flux in response to DNA damage (Fig. 1D). Moreover, we corroborated these observations in *Atg16l1*-knockout ModeK IECs using CRISPR/Cas9 gene editing. Here, we utilized our previously established model of RER by siRNA silencing of *Rnaseh2b* in *Atg16l1*-CRISPR-KO ModeK cells. We indeed confirmed that siRNA silencing of *RNaseh2b*-induced autophagy, which required functional ATG16L1 (Fig. 1E). In turn, we assessed the role of ATG16L1-dependent autophagy on the DNA-damage response in IECs, as determined by the Comet-assay. Notably, we observed that *Atg16l1*-deficient IECs evoked DNA damage (Fig. 1F, G). Collectively, these findings indicate an intestinal epithelial interplay between ATG16L1-mediated autophagy and the DNA damage response.

### Epithelial *Atg16l1* is required for the DNA damage response and controls proliferation

**Fig. 2:**
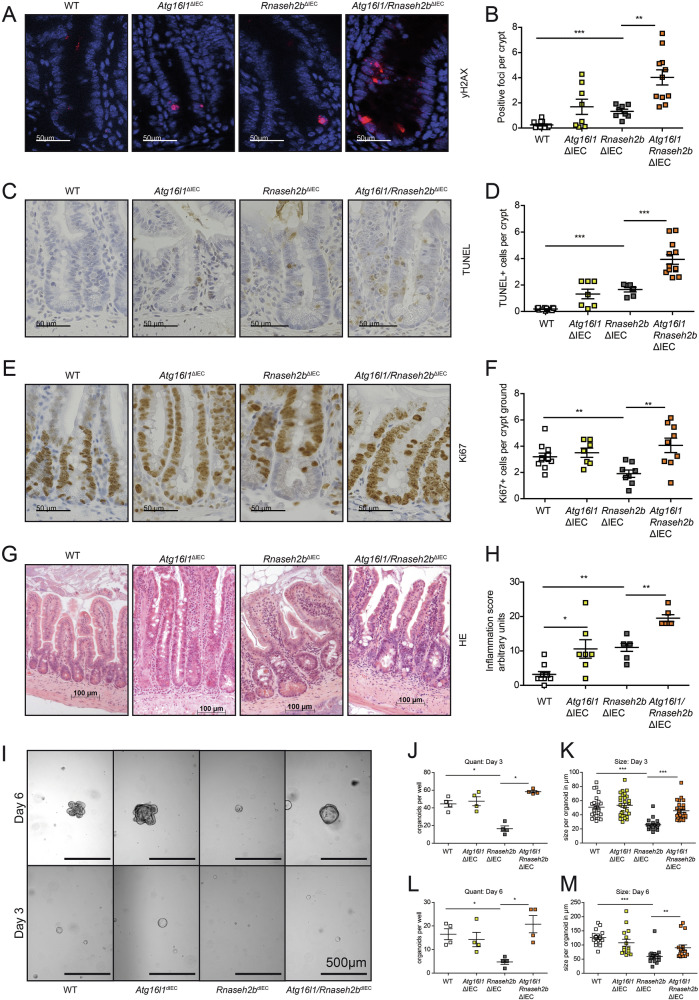
*Atg16l1* critically coordinates the epithelial DNA damage response and suppresses epithelial proliferation. **A**–**F** Immunohistology data and representative pictures of the small intestine of young (8 – 20 weeks) WT, *Atg16l1*^ΔIEC^, *Rnaseh2b*^ΔIEC^, and *Atg16l1/Rnaseh2b*^ΔIEC^ mice, stained for γ-H2Ax, TUNEL and Ki-67. Representative images (**A**) of γ-H2AX immunofluorescence staining (red) and corresponding statistics (**B**) of WT (*n* = 12; sex: 7 females, 5 males), *Atg16l1*^ΔIEC^ (*n* = 8; sex: 5 females, 3 males), *Rnaseh2b*^ΔIEC^ (*n* = 8; sex: 4 females, 4 males), and *Atg16l1/Rnaseh2b*^ΔIEC^ (*n* = 11, sex: 8 females, 3 males) mice display increased γ-H2AX positive foci per crypt in *Atg16l1/Rnaseh2b*^ΔIEC^ mice. Representative images (**C**) of TUNEL immunohistochemistry staining (brown) and corresponding statistics (**D**) of WT (*n* = 13; sex: 6 females, 7 males), *Atg16l1*^ΔIEC^ (*n* = 7; sex: 4 females, 3 males), *Rnaseh2b*^ΔIEC^ (*n* = 6; sex: 2 females, 4 males), and *Atg16l1/Rnaseh2b*^ΔIEC^ (*n* = 12; sex: 8 females, 4 males) mice display increased intestinal epithelial cell death in *Atg16l1/Rnaseh2b*^ΔIEC^ mice. Representative images (**E**) of Ki-67 immunohistochemistry staining (brown) and corresponding statistics of WT (*n* = 11; sex: 6 females, 5 males), *Atg16l1*^ΔIEC^ (*n* = 7; sex: 4 females, 3 males), *Rnaseh2b*^ΔIEC^ (*n* = 8; sex: 3 females, 5 males), and *Atg16l1/Rnaseh2b*^ΔIEC^ (n = 9; sex: 6 females, 3 males) mice display retained proliferation in *Atg16l1/Rnaseh2b*^ΔIEC^ mice in the intestinal stem cell compartment. Enteritis histology score of the small intestine. Representative images (**G**) of HE staining of the small intestine and corresponding statistics (**H**) of WT (*n* = 10; sex: 5 females, 5 males), *Atg16l1*^ΔIEC^ (*n* = 7; sex: 3 females, 4 males), *Rnaseh2b*^ΔIEC^ (*n* = 8; sex: 4 females, 4 males), and *Atg16l1/Rnaseh2b*^ΔIEC^ (*n* = 6; sex: 3 females, 3 males) mice display increased inflammation in *Atg16l1/Rnaseh2b*^ΔIEC^ mice. The Scale bar represents as indicated either 50 or 100 μm. Each data point corresponds to one animal, 50 crypts were evaluated per animal. Data are shown as mean ± SEM and were tested for statistical significance using non-parametric Mann-Whitney U test. Small intestinal organoids were generated from WT, *Atg16l1*^ΔIEC^, *Rnaseh2b*^ΔIEC^, and *Atg16l1/Rnaseh2b*^ΔIEC^ mice. Representative pictures (**I**) were taken on day three and day six after single cells were seeded out. The quantity of formed organoids per well (*n* = 4) was determined on day three (**J**) and six (**K**). The diameter of the biggest organoids of each well was determined on day three (**L**), including WT (*n* = 26), *Atg16l1*^ΔIEC^ (*n* = 19), *Rnaseh2b*^ΔIEC^ (*n* = 27), and *Atg16l1/Rnaseh2b*^ΔIEC^ (*n* = 27) organoids. The diameter of the biggest organoids in each well was determined on day six (M) including WT (*n* = 16), *Atg16l1*^ΔIEC^ (n = 16), *Rnaseh2b*^ΔIEC^ (*n* = 15), and *Atg16l1/Rnaseh2b*^ΔIEC^ (*n* = 16) organoids. This experiment was performed independently 3 times. Data are shown as mean ± SEM and were tested for statistical significance using non-parametric Mann-Whitney U test. For all the significance analysis: ns = not significant, * *p* < 0.05, ** *p* < 0.01, *** *p* < 0.001, **** *p* < 0.0001.

To study the impact of epithelial ATG16L1-dependent autophagy on DNA damage in vivo, we generated conditional double knockout mice for *Atg16l1* and *Rnaseh2b* (termed hereafter: *Atg16l1/Rnaseh2b*^ΔIEC^ mice). Wildtype mice (hereafter: *Atg16l1/Rnaseh2b*^fl/fl^ mice) and single knockout animals (hereafter: *Atg16l1*^ΔIEC^ and *Rnaseh2b*^ΔIEC^ mice) served as controls. IEC death and inhibition of epithelial proliferation are phenotypic hallmarks of chronic DNA damage in *Rnaseh2b*^ΔIEC^ mice, linking DNA damage to epithelial stem cell function [6]. We used this model to study the function of autophagy during intestinal epithelial DNA damage, which we phenotyped in young (8-20 weeks) and aged (40-60 weeks) *Rnaseh2b*^ΔIEC^ and *Atg16l1/Rnaseh2b*^ΔIEC^ mice. First, we observed that epithelial *Atg16l1* deletion significantly increased epithelial DNA damage induced by *Rnaseh2b* deletion, as indicated by γ-H2AX labeling (Fig. 2A, B). Of note, we observed in some *Atg16l1*^ΔIEC^ mice indications of spontaneous DNA damage, which was however statistically not significant and might simply display a concomitant feature of spontaneous enteritis. We further noted that epithelial deletion of *Atg16l1* increased epithelial cell death, as indicated by TUNEL labeling in *Atg16l1/Rnaseh2b*^ΔIEC^ mice compared to *Rnaseh2b*^ΔIEC^ (Fig. 2C, D). More importantly, *Atg16l1/Rnaseh2b*^ΔIEC^ mice presented abolished epithelial proliferative arrest, as indicated by Ki-67 labeling (Fig. 2E, F). This phenotype in *Atg16l1/Rnaseh2b*^ΔIEC^ mice was associated with pronounced spontaneous chronic enteritis (Fig. 2G, H). To investigate whether increased intestinal epithelial proliferation is an epithelial-intrinsic mechanism, we performed organoid colony forming assays with indicated genotypes. Indeed, we observed a significant increase in organoid colonies Fig. 2I, J, L) and enlarged organoids (Fig. 2K, M) derived from *Atg16l1/Rnaseh2b*^ΔIEC^ compared to organoids from *Rnaseh2b*^ΔIEC^ mice. These data indicate that epithelial ATG16L1 protects against DNA damage and contributes to the suppression of epithelial proliferation under conditions of increased DNA damage.

### DNA damage in *Atg16l1/Rnaseh2b*^ΔIEC^ mice does not instigate spontaneous carcinogenesis

**Fig. 3:**
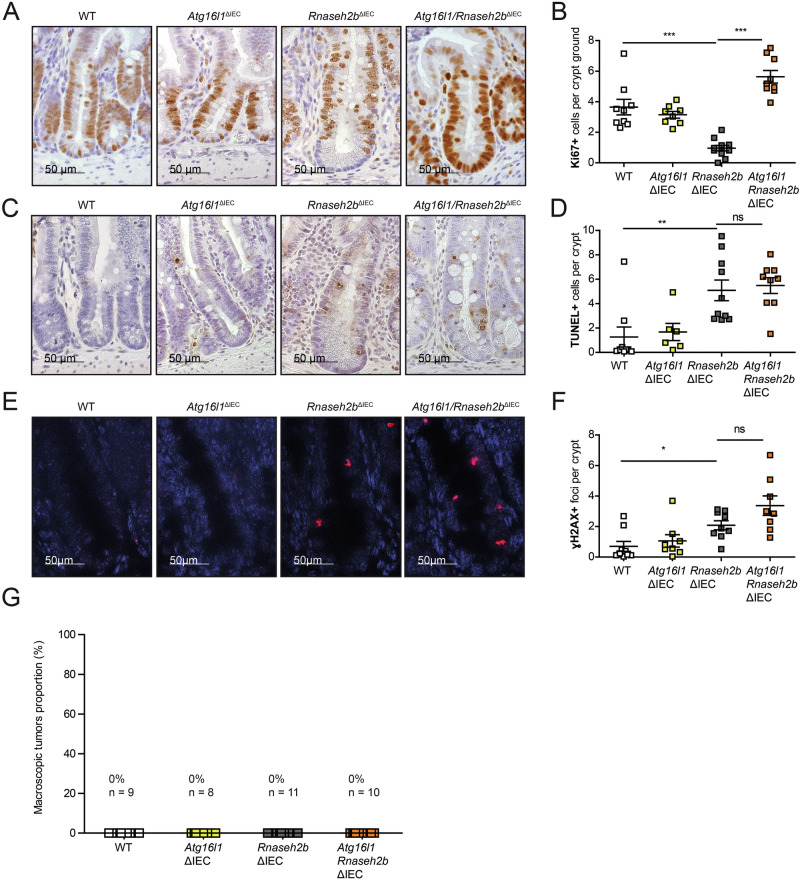
Increased proliferation in aged *Atg16l1/Rnaseh2b*^ΔIEC^ mice did not lead to intestinal tumors. **A**–**F** Immunohistology data and representative pictures of the small intestine of aged (40 – 62 weeks) of WT, *Atg16l1*^ΔIEC^, *Rnaseh2b*^ΔIEC^, and *Atg16l1/Rnaseh2b*^ΔIEC^ mice stained for γH2Ax, TUNEL, and Ki-67. Representative images (**A**) of Ki-67 immunohistochemistry staining (brown) and corresponding statistics (**B**) of WT (*n* = 9; sex: 5 females, 4 males), *Atg16l1*^ΔIEC^ (*n* = 8; sex: 5 females, 3 males), *Rnaseh2b*^ΔIEC^ (*n* = 11; sex: 2 females, 9 males), and *Atg16l1/Rnaseh2b*^ΔIEC^ (*n* = 9; sex: 9 males) mice. Representative images (**C**) of TUNEL immunohistochemistry staining (brown). And corresponding statistics (**D**) of WT (*n* = 9; sex: 5 females, 4 males), *Atg16l1*^ΔIEC^ (*n* = 6; sex: 3 females, 3 males), *Rnaseh2b*^ΔIEC^ (*n* = 10; sex: 2 females, 8 males), and *Atg16l1/Rnaseh2b*^ΔIEC^ (*n* = 9; sex: 9 males) mice. Representative images (**E**) of γ-H2AX immunofluorescence staining (red) and corresponding statistics (**F**) of WT (*n* = 9; sex: 5 females, 4 males), *Atg16l1*^ΔIEC^ (*n* = 8; sex: 4 females, 4 males), *Rnaseh2b*^ΔIEC^ (*n* = 10; sex: 2 females, 8 males), and *Atg16l1/Rnaseh2b*^ΔIEC^ (*n* = 9; sex: 9 males) mice. The scale bar represents either 50 or 100 μm. Each data point corresponds to one animal; 50 crypts were evaluated per animal. **G** None of the aged mice presented with tumors after macroscopic assessment of the whole intestine. For comparison, data from Aden et. al (*Rnaseh2b/Trp53*^ΔIEC^) and Welz et. al (*Xbp1/Rnaseh2b*^ΔIEC^) were included. Data are shown as mean ± SEM and were tested for statistical significance using non-parametric Mann-Whitney U test. ns = non-significant, * *p* < 0.05, ** *p* < 0.01, *** *p* < 0.001.

Abrogated epithelial DNA damage response, meaning retained epithelial proliferation despite ongoing DNA damage, may evoke spontaneous carcinogenesis. In previous studies using the *Rnaseh2b*^ΔIEC^ mouse model, we demonstrated that dysfunction of both the p53 and *Xbp1* pathways critically contributes to spontaneous intestinal inflammation and carcinogenesis through a mechanism of unrestrained epithelial proliferation. Notably, this phenotype develops in an age-dependent manner with manifestation of tumors at an age of approximately 52 weeks. To investigate whether impaired DNA damage response in *Atg16l1/Rnaseh2b*^ΔIEC^ evokes spontaneous intestinal carcinogenesis, we assessed overall survival and spontaneous tumorigenesis in mice that were aged up to 52 weeks. Using Ki-67 staining of the small intestine, we could observe excessive epithelial proliferation in *Atg16l1/Rnaseh2b*^ΔIEC^, which was significantly augmented compared to *Rnaseh2b*^ΔIEC^ or *Atg16l1*^ΔIEC^ mice (Fig. 3A, B). Interestingly, we also observed increased cell death using TUNEL staining (Fig. 3C, D) and DNA damage using γ-H2AX staining (Fig. 3E, F), but these were not significantly increased in *Atg16l1/Rnaseh2b*^ΔIEC^ compared to *Rnaseh2b*^ΔIEC^ mice. However, in contrast to the loss of proliferative arrest, we surprisingly found that aged *Atg16l1/Rnaseh2b*^ΔIEC^ mice did not develop spontaneous intestinal tumors (Fig. 3G).

### Loss of epithelial *Atg16l1* does not impair p53-driven DNA damage response

**Fig. 4:**
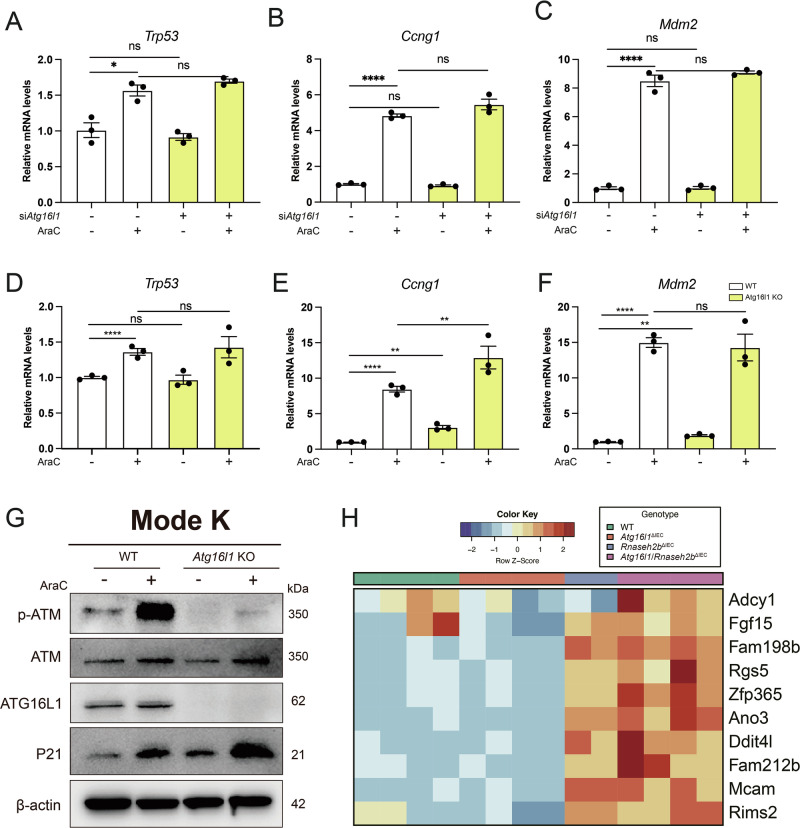
*Atg16l1* deficiency did not impair p53 response to DNA damage. **A**–**C** Mode-K cells were transfected with either siRNA against *Atg16l1* or control siRNA and stimulated for 24 h with 2.5 μM AraC. Gene expression of *Trp53*, *Ccng1*, *Mdm2* validated by Taqman assay. **D**–**F** Mode-K WT or *Atg16l1* knockout cells stimulated for 24 h with 2.5 μM AraC. Gene expression of *Trp53*, *Ccng1*, *Mdm2* validated by Taqman assay. Representative of a minimum of 3 individual experiments with 3 technical replicates. Data are shown as mean with ± SEM and were tested for statistical significance using unpaired Student’s *t*-test. **G** Mode-K WT or *Atg16l1* knockout cells stimulated for 24 h with 2.5 μM AraC, protein levels measured by Western Blot assay. **H** RNA-sequencing analysis in organoids isolated from WT*, Atg16l1*^ΔIEC^*, Rnaseh2b*^ΔIEC^*, Rnaseh2b/Atg16l1*^ΔIEC^ mice, p53 pathway related genes were clustered using a heatmap. Color code base on z-score. For all the significance analysis: ns = not significant, * *p* < 0.05, ** *p* < 0.01, *** *p* < 0.001, **** *p* < 0.0001.

To investigate whether protection from intestinal carcinogenesis might be established due to a retained p53 response in autophagy-deficient intestinal epithelial cells, we assessed the induction of *Trp53* and downstream p53 target genes *Ccng1, Mdm2*. We first used ModeK cells silenced with siRNA (*Atg16l1* vs. Ctrl) and subsequent AraC treatment. In line with previous findings, AraC treatment induced *Trp53, Ccng1* and *Mdm2* expression upon DNA damage, which was comparable in ModeK cells with *Atg16l1* knockdown by siRNA (Fig. 4A–C). We confirmed this finding in *Atg16l1*-KO ModeK cells, in which we observed a similar activation of the p53 response compared to wildtype ModeK cells (Fig. 4D–F). The retained p53 response was further confirmed at the protein level, showing that AraC treatment led to, if anything, increased p21 protein expression in *Atg16l1*-KO ModeK cells (Fig. 4F). Interestingly, we observed decreased phosphorylation of ATM in *Atg16l1*-KO ModeK, which might point towards a direct interaction of ATG16L1 with the coordination of DNA damage responses (Fig. 4G). Lastly, we conducted RNA sequencing from intestinal organoids (*Rnaseh2b*^*fl/fl*^*, Atg16l1*^ΔIEC^*, Rnaseh2b*^ΔIEC^ *and Atg16l1/Rnaseh2b*^ΔIEC^). To understand whether ATG16L1 deficiency affects p53 response in the context of the genetic model (*Atg16l1/Rnaseh2b*^ΔIEC^ vs. *Rnaseh2b*^ΔIEC^), we particularly assessed the expression of genes that we previously described to be uniquely regulated via p53, including *Ddit4l*. In line with previous findings, we observed upregulation of the genes *Ano3, Fam212b* and *Ddit4L* in *Rnaseh2b*^ΔIEC^ and in *Atg16l1/Rnaseh2b*^ΔIEC^ organoids (Fig. 4H). Hence, our data indicate that the impairment of autophagy does not affect the p53 response in IEC, which might explain the loss of tumor manifestation in *Atg16l1/Rnaseh2b*^ΔIEC^ mice.

### Epithelial deficiency of *Atg16l1* and *Xbp1* cooperatively fuel DNA damage

In earlier studies, we have shown that *Xbp1/Rnaseh2b*^ΔIEC^ exhibit increased intestinal epithelial DNA damage as well as increased stem cell proliferation compared to *Rnaseh2b*^ΔIEC^ mice, and 48% of these mice develop intestinal tumor,s which are largely adenocarcinomas [15]. As shown before, *Atg16l1/Rnaseh2b*^ΔIEC^ mice showed increased intestinal epithelial DNA damage as well as increased stem cell proliferation. However, in contrast to *Xbp1/Rnaseh2b*^ΔIEC^ mice, *Atg16l1/Rnaseh2b*^ΔIEC^ mice do not spontaneously develop intestinal adenocarcinomas. As the compensatory interplay between ATG16L1-mediated autophagy and ER stress resolution have been shown to be crucially involved in Paneth cell homeostasis in the disease pathophysiology of small intestinal inflammation and likely CD [8], we wondered whether this effect might also play a role in DNA damage-induced stem cell function and carcinogenesis. To test this hypothesis, we generated triple knockout mice carrying an epithelial-specific deletion of *Atg16l1*, *Xbp1* and *Rnaseh2b* (hereafter: *Atg16l1/Xbp1/Rnaseh2b*^ΔIEC^ mice). Again, we compared the degree of DNA damage (γ-H2AX), epithelial cell death (TUNEL) and epithelial proliferation (Ki-67, BrdU) in young (8-15 weeks) *Atg16l1/Xbp1/Rnaseh2b*^ΔIEC^ mice and compared their phenotype to double mutant controls (i.e., *Xbp1/Rnaseh2b*^ΔIEC^, *Atg16l1/Rnaseh2b*^ΔIEC^) or single knockout mice (*Rnaseh2b*^ΔIEC^). We observed that young triple knockout mice exhibited augmented DNA damage, IEC death and even higher increase of epithelial proliferation in the small intestinal epithelial crypt region (Fig. S1).

### Epithelial deficiency of *Atg16l1* and *Xbp1* promotes DNA damage-induced carcinogenesis by impairing Ddit4l

**Fig. 5:**
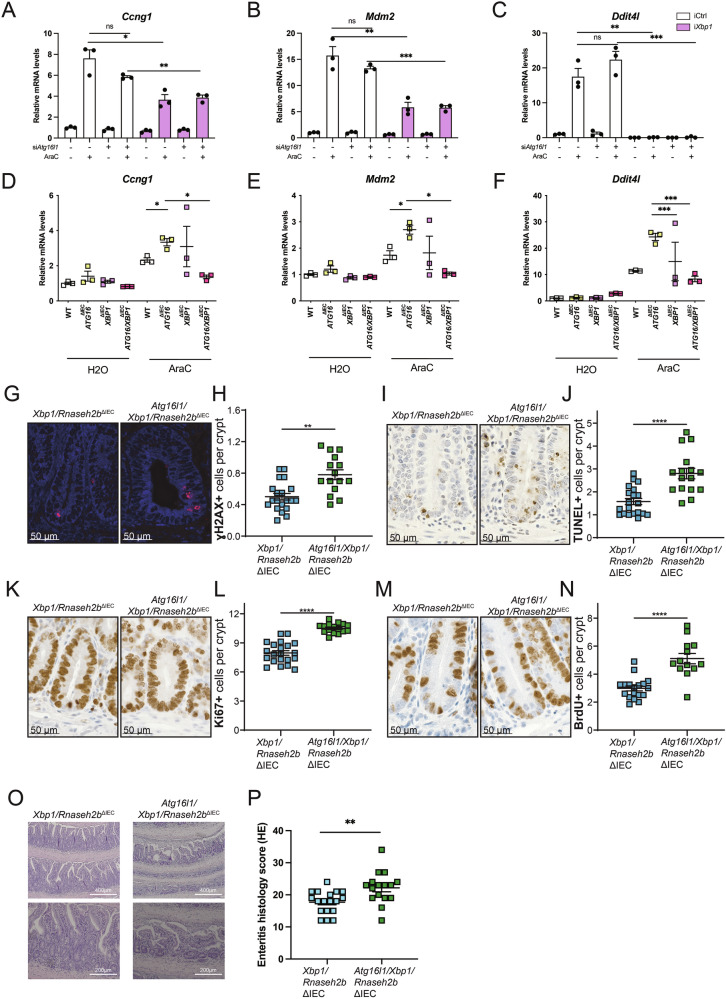
Cooperative effects of ATG16L1 and XBP1 deficiency in promoting DNA damage response and proliferation. **A**–**C** Mode-K cells with Xbp1 deficiency treated with 2,5 μM AraC for 25 h, gene expression of *Ccng1*, *Mdm2*, *Ddit4l* measured by Taqman assay. Representative of a minimum of 3 individual experiments with 3 technical replicates. **D**–**F** SI organoids isolated from WT*, Atg16l1*^ΔIEC^*, Xbp1*^ΔIEC^*, Atg16l1/Xbp1*^ΔIEC^ mice stimulated with 2.5 μM AraC for 24 h, gene expression of *Ccng1*, *Mdm2*, *Ddit4l* measured by Taqman assay. Representative of a minimum of 3 individual experiments with 3 technical replicates. Data are shown as mean with ± SEM and were tested for statistical significance using unpaired Student’s *t*-test. **G–P** Immunohistology data and representative pictures of aged (52 weeks – 72 weeks) *Xbp1/Rnaseh*2b^ΔIEC^ (*n* = 18; Sex: 9 females, 9 males; Age: Ø = 62 weeks) and *Atg16l1/Xbp1/Rnaseh2b*^ΔIEC^ (*n* = 16; Sex: 7 females, 9 males; Age: Ø = 58 weeks) mice. Small intestinal epithelium stained for γH2Ax (G, H), TUNEL (**I**, J), Ki-67 (**K**, **L**) and BrdU (**M**, **N**). For BrdU staining, fewer mice were analyzed since the required in vivo BrdU injection was not performed. Each data point corresponds to one animal, 24 crypts were evaluated per animal. In every crypt, the bottom 12 cells were accessed. Data are expressed as mean with standard error of the mean. Significance was determined using Mann-Whitney-Test. **O**, **P** Enteritis histology score of the small intestine. Representative images (**O**) of the small intestine and corresponding statistics (P). For all the significance analysis: ns = not significant, * *p* < 0.05, ** *p* < 0.01, *** *p* < 0.001, **** *p* < 0.0001.

We next tested on the molecular level whether co-deletion of *Atg16l1* and *Xbp1* impairs Ddit4l expression in response to DNA damage by using two in vitro models. First, we manipulated ModeK cells (WT vs. i*Xbp1*) with siRNA (*Atg16l1* vs. Ctrl) to generate double-deficient ModeK cells for *Atg16l1* and *Xbp1* and stimulated the cells with AraC. We observed that AraC-induced expression of the p53 target genes *Ccng1, Mdm2* and *Ddit4l* was significantly reduced in *Atg16l1*/*Xbp1* double-deficient ModeK cells (Fig. 5A–C). In addition, we stimulated murine small intestinal organoids (WT, *Atg161l*^*ΔIEC*^*, Xbp1*^*ΔIEC*^*, Atg16l1/Xbp1*^*ΔIEC*^) with or without AraC and again assessed the expression of *Ccng1, Mdm2* and *Ddit4l*. Thus, we confirmed at the molecular level the reduced p53-response in *Atg16l1*/*Xbp1* double-deficient intestinal organoids (Fig. 5D–F). To further investigate the consequences in vivo, we aged *Atg16l1/Xbp1/Rnaseh2b*^ΔIEC^ mice (and respective controls) up to 72 weeks. *Atg16l1/Xbp1/Rnaseh2b*^ΔIEC^ mice retained the epithelial phenotype in terms of epithelial DNA damage (Fig. 5G, H), IEC death (Fig. 5I, J) and increased Ki-67 (Fig. 5K, L) and BrdU-staining (Fig. 5I, M, N) when compared to and *Xbp1/Rnaseh2b*^ΔIEC^ mice. In addition, we observed increased small intestinal enteritis score in *Atg16l1/Xbp1/Rnaseh2b*^ΔIEC^, compared to *Xbp1/Rnaseh2b*^ΔIEC^ (Fig. 5O, P).

**Fig. 6:**
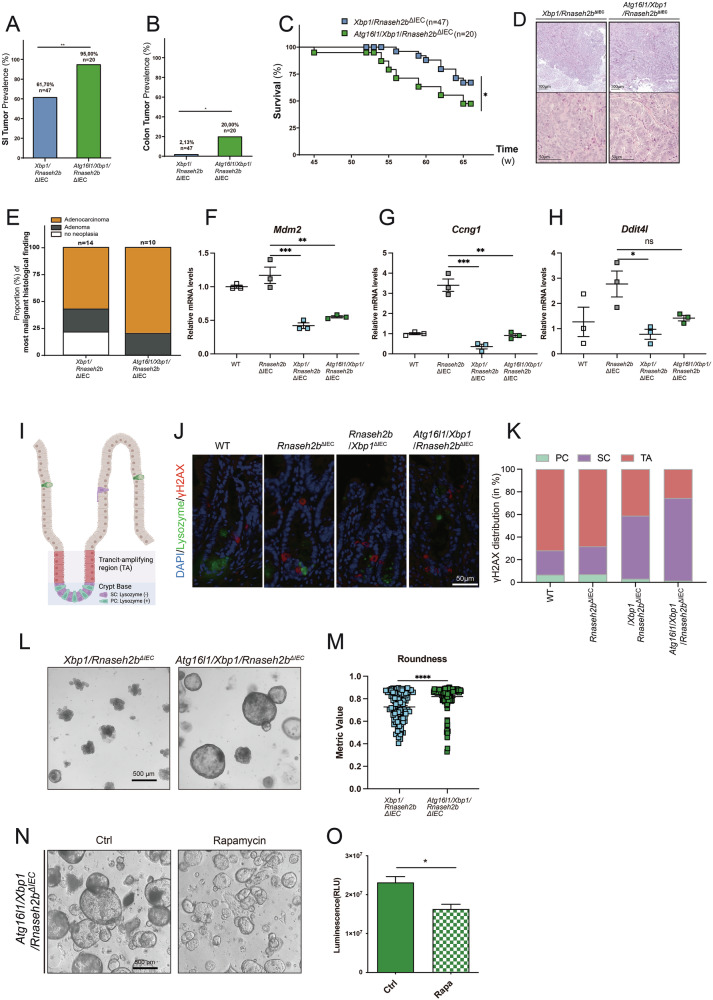
*Atg16l1* deficiency fuels induction of intestinal adenocarcinoma. **A**, **B** Prevalence of small intestinal (**I**) and colonic (**J**) tumors in aged (52 weeks–72 weeks) *Xbp1/Rnaseh2b*^ΔIEC^ (*n* = 47; sex: 26 females, 21 males; Age: Ø = 59,2 weeks) and *Atg16l1/Xbp1/Rnaseh2b*^ΔIEC^ (*n* = 20; sex: 10 females, 10 males; Age: Ø = 58,5 weeks) mice. Significance was determined using a two-sided Fisher’s exact test: ns = not significant. **C** Survival (K) of aged (max. 65 weeks) *Xbp1/Rnaseh2b*^ΔIEC^ (*n* = 21; Sex: 9 females, 12 males; Age: Ø=64,7 weeks) and *Atg16l1/Xbp1/Rnaseh2b*^ΔIEC^ (*n* = 13; Sex: 8 females, 5 males; Age: Ø=61,7 weeks) mice. Significance was determined using the log-rank test based on Kaplan-Meier analysis. Histopathological grading of small intestinal tumors of aged (52 - 72 weeks) *Xbp1/Rnaseh2b*^ΔIEC^ (*n* = 14; Sex: 5 females, 9 males; Age: Ø = 65,6 weeks) and *Atg16l1/Xbp1/Rnaseh2b*^ΔIEC^ (*n* = 10; Sex: 5 females, 5 males; Age: Ø = 63,3 weeks) mice: Grading of the most malignant histological finding per mouse (**D**) and representative pictures (**E**) of stated histopathological grading (HE). **F**–**H** The gene expressions of *Ccng1*, *Mdm2*, and *Ddit4l* in the small intestine tissues of aged WT*, Rnaseh2b*^ΔIEC^*, Rnaseh2b/Atg16l1*^ΔIEC^*, Atg16l1/Xbp1/Rnaseh2b*^ΔIEC^ mice (*n* = 3 each genotype) were detected by Taqman assay. Data are shown as mean with ± SEM. Significance was determined using an unpaired student’s *t* test. **I**–**K** Co-immunofluorescence staining of γH2AX and lysozyme was performed on intestinal sections from WT (*n* = 6)*, Rnaseh2b*^*ΔIEC*^ (*n* = 6)*, Rnaseh2b/Atg16l1*^ΔIEC^ (*n* = 6)*, Atg16l1/Xbp1/Rnaseh2b*^ΔIEC^ mice (*n* = 5). Crypt regions were segmented into transit-amplifying (TA) zone and basal zone. Lysozyme expression was used to distinguish Paneth cells (lysozyme +) from intestinal stem cells (lysozyme-) in the basal region. Crypts exhibiting γH2AX+ nuclei were quantified. The proportions of γH2AX+ cells within each cell type and zone were calculated based on the total number of cells analyzed. Scale bar = 50 μm. **L**, **M** Roundness analysis from organoids. Significance of was determined using unpaired student’s t test. N-O: (N) CellTiter-Glo assay (**O**) and representative images (N) (scale bar = 500 μm) of *Atg16l1*/*Xbp1*/*Rnaseh2b*^ΔIEC^ small-intestinal organoids stimulated with Control (DMSO) or 1 μmol/L rapamycin for 72 h. Representative of a minimum of 3 individual experiments with 3 technical replicates. Data are shown as mean with ± SEM. Significance of was determined using unpaired student’s *t* test. For all the significance analysis: ns = not significant, *p < 0.05, **p < 0.01, ***p < 0.001, ****p < 0.0001.

More importantly, we noted that aged *Atg16l1/Xbp1/Rnaseh2b*^ΔIEC^ mice spontaneously presented with nearly complete penetrance of small intestinal tumors (95.0%), when compared to *Xbp1/Rnaseh2b*^ΔIEC^ mice (61.7%) (Fig. 6A). Although differences in the colon were less pronounced, we also noted colonic tumors in aged *Atg16l1/Xbp1/Rnaseh2b*^ΔIEC^ mice (20.0%), when compared to aged *Xbp1/Rnaseh2b*^ΔIEC^ mice (2.13%) (Fig. 6B). Increased prevalence of small intestinal and colonic tumors resulted in decreased survival of *Atg16l1/Xbp1/Rnaseh2b*^ΔIEC^ mice (Fig. 6C). A group of 14 *Xbp1/Rnaseh2b*^ΔIEC^ mice and 10 *Atg16l1/Xbp1/Rnaseh2b*^ΔIEC^ mice were histopathologically analyzed, which confirmed that both genotypes largely developed adenocarcinomas (Fig. 6D, E). Adenocarcinomas from *Atg16l1/Xbp1/Rnaseh2b*^ΔIEC^ mice were not more dedifferentiated than those from *Xbp1/Rnaseh2b*^ΔIEC^ mice. In fact, mice with functional autophagy even displayed a tendency to develop more dedifferentiated carcinomas, as we found more high-grade adenocarcinomas in *Xbp1/Rnaseh2b*^ΔIEC^ mice compared to *Atg16l1/Xbp1/Rnaseh2b*^ΔIEC^ mice (Fig. 6D, E).

We further confirmed decreased p53 response in *Atg16l1/Xbp1/Rnaseh2b*^ΔIEC^ via qRT-PCR of small intestinal tissue section, where we observed significant reduction in p53 pathway genes while *Ddit4l* level also reduced (*p* = 0.06) (Fig. 6F–H).

To investigate how DNA damage differentially affects distinct cell types within the intestinal crypt, we conducted co-staining of γH2AX and lysozyme. Compared to WT and *Rnaseh2b*^ΔIEC^, *Atg16l1*/*Rnaseh2b*^ΔIEC^ mice exhibited predominant γ-H2AX⁺ nuclei in the basal zone of the crypts. Among crypt basal cells, lysozyme-negative intestinal stem cells showed higher levels of γ-H2AX staining, while very few lysozyme⁺ Paneth cells were γ-H2AX-positive, suggesting that stem cells exhibit heightened DNA damage responses. This pattern was especially pronounced in the *Atg16l1*/*Xbp1*/*Rnaseh2b*^ΔIEC^ group, highlighting a synergistic role of ATG16 L1 deficiency in damaging the stem cell compartment (Fig. 6I–K). To recapitulate the observed in vivo phenotype in vitro, we generated intestinal organoids from indicated genotypes and conducted an automated roundness analysis score, in which we observed significantly increased roundness as a metric of unrestrained proliferation in *Atg16l1/Xbp1/Rnaseh2b*^ΔIEC^-derived organoids (Fig. 6L, M). Lastly, we tested the hypothesis that the loss of *Ddit4l* expression in *Atg16l1/Xbp1/Rnaseh2b*^ΔIEC^ mice might provide a therapeutic intervention point. We therefore stimulated *Atg16l1/Xbp1/Rnaseh2b*^ΔIEC^-derived organoids with or without rapamycin. Using CellTiter-Glo assay, we observed that rapamycin led to a significant reduction of organoid survival (Fig. 6N, O). Altogether, these data indicate that the loss of *Xbp1* and *Atg16l1* synergistically contributes to the initiation of intestinal carcinogenesis driven by RER deficiency. This effect is to a great extent mediated via the suppression of *Ddit4l*, which highlights the role of the mTOR pathway in this genetic model of transcription-associated mutagenesis.

## Discussion

Small bowel adenocarcinoma (SBA) is a rare long-term disease complication of IBD patients with currently limited therapeutic options. The clear association of SBA incidence with the presence of complicating (structuring, penetrating) IBD phenotype underscores a putative function of disease-relevant pathophysiological pathways in the malignant transformation. Transcription-associated mutagenesis describes a mechanism of the constant acquisition of DNA mutations due to misled incorporation of ribonucleotides into the genome. Upon dysfunction of RNase H2, compensatory TOP1-mediated DNA repair thereby instigates indel (insertion-deletion mutations) formation, which generates a mutational susceptibility landscape for carcinogenesis [29]. Chronic inflammation provides an essential driving force of transcription-associated mutagenesis as high epithelial turnover is needed to cope with barrier breaches in the intestinal mucosa. In this study, we assessed the interplay of two well-described IBD risk genes, *Atg16l1* and *Xbp1*, in the coordination of DNA repair in the intestinal epithelium. We demonstrated that DNA damage, induced either genetically by *Rnaseh2b* deletion or chemically by AraC treatment, activates autophagy in the intestinal epithelium in an ATG16L1-dependent manner. In mice, inhibition of autophagy through *Atg16l1* deletion increases DNA damage caused by defective ribonucleotide excision repair (RER) in the intestinal epithelium, as evidenced by γ-H2AX staining in mice and comet-assays in vitro, the latter providing a more direct measure of accumulated DNA damage at the cellular level. These findings underscore that autophagy has a key regulatory role in the initial DNA damage responses in the intestinal epithelium. This proliferative arrest in intestinal stem cells with defective RER was abolished upon epithelial deletion of *Atg16l1*. Instead of the proliferative arrest, we observed drastically increased levels of epithelial cell death, especially in the small intestine. Our data thereby strongly indicate that the increased epithelial proliferation was at least partially driven by effects of the epithelial intrinsic *Atg16l1* deletion underscoring the crucial role of autophagy-dependent stem cell control in response to DNA damage. As mentioned before, key molecular events for the development of invasive lesions of the intestine, such as chromosomal instability (CIN), microsatellite instability (MSI), CpG island methylator phenotype (CIMP) and mutations in driver genes such as *APC, KRAS, TP53, PIK3CA*, and *SMAD4*, are present in the development of sporadic CRC as well as inflammation-driven CRC. What distinguishes the two types is the differing timing of these events and the relative impact, as illustrated by mutations in the *TP53* tumor suppressor, which are found in nearly 70% of IBD-associated SBA patients [13]. Notably, in both double knockout mice, such as in *Atg16l1/Rnaseh2b*^ΔIEC^ and *Xbp1/Rnaseh2b*^ΔIEC^ mice, we simultaneously observed increased proliferation and cell death. However, deletion of *Atg16l1* and *Rnaseh2b* was not sufficient to elicit age-dependent spontaneous carcinogenesis, as it has been shown previously for *Xbp1/Rnaseh2b*^ΔIEC^ and *Trp53/Rnaseh2b*^ΔIEC^. Thus, dysfunctional autophagy alone is not enough to drive DNA-damaged cells into malignant transformation of *Atg16l1/Rnaseh2b*^ΔIEC^ mice. In line with this finding, we observed that p53-dependent transcriptional activity was still retained in *Atg16l1/Rnaseh2b*^ΔIEC^ IECs, which putatively explains the lack of malignant transformation. Following the hypothesis of cross-regulatory compensation of autophagy and ER stress, which has already been shown in the context of small intestinal inflammation, we assessed this interplay in the context of cancer development. We surprisingly found that the deletion of *Atg16l1* led to a significantly increased tumor prevalence in *Atg16l1/Xbp1/Rnaseh2b*^ΔIEC^ mice (no functional autophagy present to compensate for defective UPR) compared to *Xbp1/Rnaseh2b*^ΔIEC^ mice (autophagy compensates for the defective UPR) of the same age. Interestingly, we observed more pronounced effects in the small intestine compared to the colon. This regional disparity is not unprecedented and has been reported in established models such as the *Apc*^Min/+^ mouse, which develops markedly more polyps in the small intestine than in the colon [30]. Several factors may underlie this difference, including regional variation in Wnt signaling activity, epithelial turnover, and stem cell distribution [31] as well as distinct microbial and immune landscapes along the gut [32]. These intrinsic differences may render the small intestine more susceptible to certain oncogenic or inflammatory stimuli and should be considered when interpreting region-specific phenotypes. At the molecular level, we provide evidence that impairment of autophagy provides an additional p53-independent tipping point to fuel the induction of adenocarcinoma in the context of transcription-associated mutagenesis and impaired p53 tumor suppression. In IBD, the interplay between *XBP1* and *ATG16L1* is crucial for maintaining ER homeostasis, and dysfunctions of this interaction can lead to cell death and severe intestinal inflammation. Our data suggest that this interplay not only substantially contributes to gut epithelial homeostasis during inflammation but is also critically involved in transcription-associated carcinogenesis.

Carcinogenesis is a highly complex process that consists of many different hallmarks. Yet, it can be simplified into 4 stages: Initiation, malignant transformation, and progression. It is widely established that autophagy has a paradoxical and dualistic impact on carcinogenesis depending on the stage of disease [33, 34]. In particular, it is important to distinguish between the effects of autophagy on the emergence of malignant cells (consisting of initiation, promotion, and malignant transformation) and the progression of already existing malignant cells. Previous studies reported that the dysfunctional Thr300Ala variant of ATG16L1 is associated with enhanced survival in patients with Crohn’s disease and CRC [35]. Numerous studies have shown that dysfunctional autophagy increases the vulnerability of both healthy and cancer cells to various forms of stress, such as ER stress [8], hypoxia [36], oxidative stress [37, 38], and chemotherapeutic treatment [39]. Furthermore, it was recently shown that dysfunctional autophagy increases programmed cell death induced by IFN-γ and TNF and consequently increases the host immunity to colorectal cancer cells. Thus, colorectal cancers with high expression of *ATG16L1* were associated with poor clinical outcomes under PD-L1 therapy [40]. Our data reveal the surprising finding that the initiation of tumorigenesis critically depends on the interplay between ER stress and autophagy; disruption of this interaction dramatically accelerates progression to invasive carcinoma. Altogether, our data provide evidence for a crucial interaction of two established IBD risk genes and pathophysiological principles (ER stress resolution, autophagy) not only in the pathogenesis of intestinal inflammation but also in the development of small intestinal carcinoma, which might provide an important actionable entry point in the prevention of small bowel carcinoma.

## Supplementary information

Supplementary figures_Figures and legends

Supplementary Table 1

Supplementary Table 2

Supplementary Table3

Supplementary Table4

## Data Availability

The raw RNA sequencing data generated in this study have been deposited in the Gene Expression Omnibus (GEO) database under accession number GSE299677. All data, analytic methods and study material will be made available for other researchers by request.
