# Supplementary material for: Atg16l1 and Xbp1 cooperatively protect from transcription-associated mutagenesis and small intestinal carcinogenesis
Source: Oncogene. 2025 Oct 7;44(45):4413–26. doi: 10.1038/s41388-025-03591-x (PMC12583144; doi:10.1038/s41388-025-03591-x)
Supplement: Supplementary file 1 — Supplementary figures_Figures and legends [file 41388_2025_3591_MOESM1_ESM.pdf]

Supplementary Figure 1: Deletion of *Atg16l1* and *Xbp1* synergistically promote small intestinal epithelial DNA damage and cell death along with increased stem cell proliferation in young mice.

Immunohistology data and representative pictures of the small intestine of young (8 - 15 weeks) *Rnaseh2b*$^{\Delta IEC}$ (n=7; sex: 3 females, 4 males), *Atg16l1/Rnaseh2b*$^{\Delta IEC}$ (n=9; sex: 7 females, 2 males), *Xbp1/Rnaseh2b*$^{\Delta IEC}$ (n= 9; sex: 3 females, 6 males) and *Atg16l1/Xbp1/Rnaseh2b*$^{\Delta IEC}$ (n=9; sex: 6 females, 3 males) mice, stained for γH2Ax (A,B), TUNEL (C,D), Ki-67 (E,F) and BrdU (G,H). For BrdU staining, less mice were analyzed since the required *in vivo* BrdU injection was not performed for every replicate. Each data point corresponds to one animal, 24 crypts were evaluated per animal. In every crypt, the bottom 12 cells were accessed. Data are expressed as mean with standard error of the mean. Significance was determined using Mann-Whitney U test.

For all the significance analysis: ns = not significant, * $p<0.05$, ** $p<0.01$, *** $p<0.001$, **** $p<0.0001$.

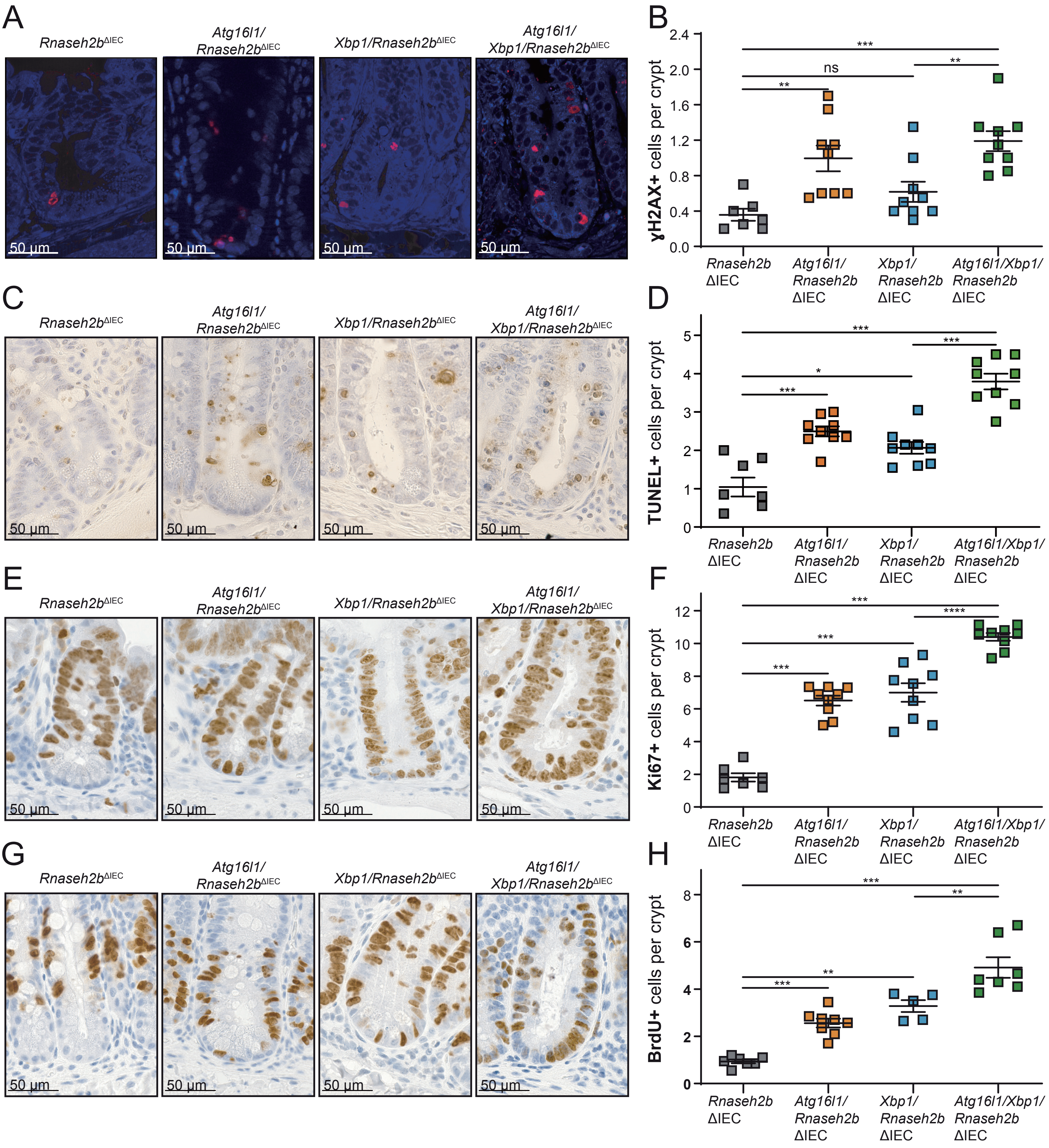

A
B
C
D
E
F
G
H
Rnaseh2bΔIEC
Atg16l1/ Rnaseh2bΔIEC
Xbp1/Rnaseh2bΔIEC
Atg16l1/ Xbp1/Rnaseh2bΔIEC
50 µm
γH2AX+ cells per crypt
TUNEL+ cells per crypt
Ki67+ cells per crypt
BrdU+ cells per crypt
Rnaseh2b ΔIEC
Atg16l1/ Rnaseh2b ΔIEC
Xbp1/ Rnaseh2b ΔIEC
Atg16l1/Xbp1/ Rnaseh2b ΔIEC
ns
